# The Effects of Adding Pictorial Depth Cues to the Poggendorff Illusion

**DOI:** 10.3390/vision6030044

**Published:** 2022-07-18

**Authors:** Gizem Y. Yildiz, Bailey G. Evans, Philippe A. Chouinard

**Affiliations:** 1 Department of Psychology, Counselling, & Therapy, La Trobe University, Melbourne 3086, Australia; g.yildiz@fz-juelich.de (G.Y.Y.); 18481164@students.latrobe.edu.au (B.G.E.); 2 Institute of Neuroscience and Medicine, INM-3, Research Center Jülich, 52425 Jülich, Germany

**Keywords:** Poggendorff illusion, pictorial depth cues, occluder size, depth processing, perceptual constancy

## Abstract

We tested if the misapplication of perceptual constancy mechanisms might explain the perceived misalignment of the oblique lines in the Poggendorff illusion. Specifically, whether these mechanisms might treat the rectangle in the middle portion of the Poggendorff stimulus as an occluder in front of one long line appearing on either side, causing an apparent decrease in the rectangle’s width and an apparent increase in the misalignment of the oblique lines. The study aimed to examine these possibilities by examining the effects of adding pictorial depth cues. In experiments 1 and 2, we presented a central rectangle composed of either large or small bricks to determine if this manipulation would change the perceived alignment of the oblique lines and the perceived width of the central rectangle, respectively. The experiments demonstrated no changes that would support a misapplication of perceptual constancy in driving the illusion, despite some evidence of perceptual size rescaling of the central rectangle. In experiment 3, we presented Poggendorff stimuli in front and at the back of a corridor background rich in texture and linear perspective depth cues to determine if adding these cues would affect the Poggendorff illusion. The central rectangle was physically large and small when presented in front and at the back of the corridor, respectively. The strength of the Poggendorff illusion varied as a function of the physical size of the central rectangle, and, contrary to our predictions, the addition of pictorial depth cues in both the central rectangle and the background decreased rather than increased the strength of the illusion. The implications of these results with regards to different theories are discussed. It could be the case that the illusion depends on both low-level and cognitive mechanisms and that deleterious effects occur on the former when the latter ascribes more certainty to the oblique lines being the same line receding into the distance.

## 1. Introduction

**Figure 1:**
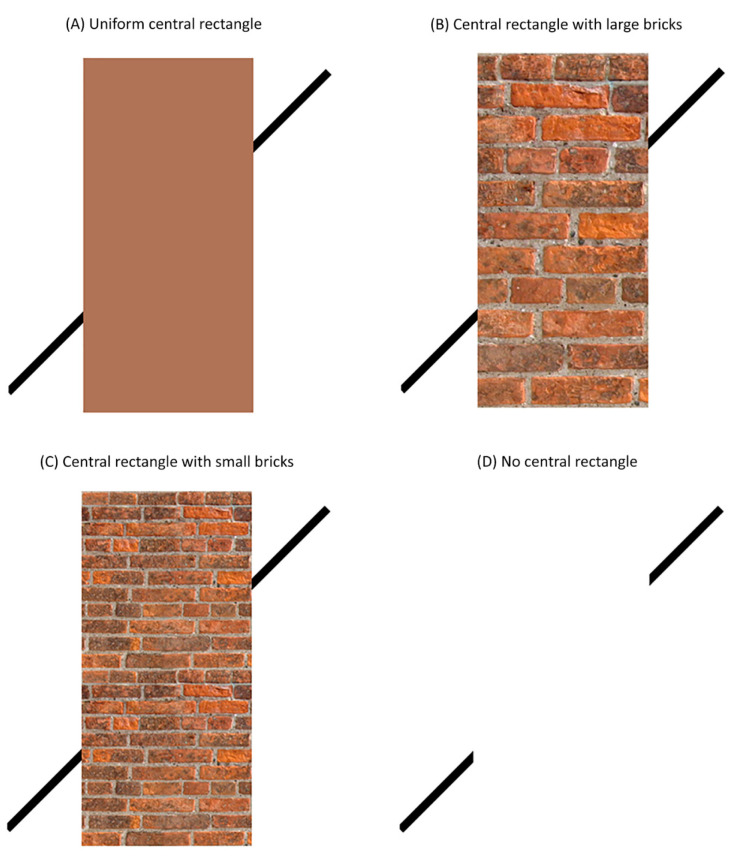
Poggendorff figures used to test for the effect of texture cues on the magnitude of the Poggendorff illusion in experiment 1. The Poggendorff stimuli consisted of (**A**) a uniform central rectangle, (**B**) a central rectangle with large bricks, (**C**) a central rectangle with small bricks and (**D**) no central rectangle. Similar central rectangles in (**A**–**C**) were used by Talasli and Inan [3].

In the Poggendorff illusion, colinear oblique lines appear to be misaligned when a rectangle is presented in the middle portion of the Poggendorff stimulus [1]. For example, in Figure 1, the upper and lower oblique lines are colinear. Yet, the upper line, on the right side of the central rectangle, appears to be placed at a position higher than the point that would be colinear to the lower line.

**Figure 2:**
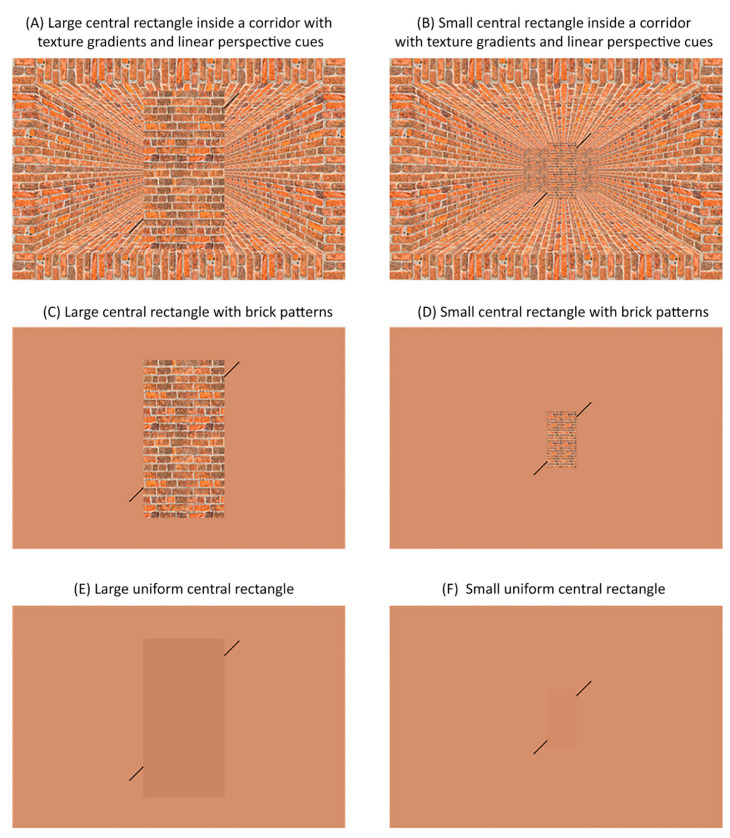
Poggendorff figures used to test for the effects of the number of pictorial depth cues and central rectangle size on the magnitude of the Poggendorff illusion in experiment 3. In experiment 3, we presented central rectangles composed of bricks in a corridor rich in texture and linear perspective depth cues (**A**,**B**), central rectangles composed of bricks on a uniform background (**C**,**D**) and central rectangles of a uniform colour on a uniform background (**E**,**F**). The central rectangles were presented either in large (**A**,**C**,**E**) or small (**B**,**D**,**F**) sizes, corresponding to positions in the front and the back of the corridor background, respectively.

Gregory [2] explained this perceived misalignment by the misapplication of mechanisms that extract depth information and alter the apparent characteristics of objects for the purposes of achieving perceptual constancy. Gregory defined perceptual constancy as the perception of an object as remaining constant as sensory information about it fluctuates, such as its size on the retina, under different conditions, such as viewing distance. Specifically, he proposed that the brain extracts depth information from the two-dimensional (2D) Poggendorff stimulus based on knowing, from previous experience, how various cues relate to depth in the real world, which is three-dimensional (3D). According to Gregory [2], there are two possible depth cues extracted from the Poggendorff stimulus. One is linear perspective whereby the oblique lines are treated as one long line receding in depth. Another is occlusion, whereby objects at nearer positions, such as the central rectangle, cover objects that are placed farther away, such as the oblique lines. Therefore, according to Gregory, the implied depth, whether it is linear perspective or occlusion, causes a perceptual distortion in the alignment of the two oblique lines in an attempt to achieve perceptual constancy. The present investigation examines the effects of pictorial depth cues on the Poggendorff illusion (Figure 1 and Figure 2).

Talasli and Inan [3] further proposed that the perceived occlusion in the Poggendorff illusion might cause the central rectangle to appear more narrow than it really is as a result of perceptual size rescaling, consequently causing an apparent shift in the positioning of the oblique lines towards the midline and making them appear more misaligned. To examine this possibility, Talasli and Inan [3] used a central rectangle composed of either large (i.e., Figure 1B) or small (i.e., Figure 1C) bricks. Because brick patterns on the retina recede with viewing distance, the authors reasoned that the central rectangle with large bricks (i.e., Figure 1B) would be treated as being closer in distance than the one with small bricks (i.e., Figure 1C) and that participants would judge the width of the central rectangle with large bricks as being more narrow than the one with small bricks. Their results supported these predictions. In addition, the authors reported that the strength of the Poggendorff illusion produced by the central rectangle with large bricks was greater than the one produced by the central rectangle with small bricks.

Corroborating Talasli and Inan’s [3] findings, Wang and Idesawa [4] demonstrated that apparent differences in depth between the central rectangle and the oblique lines can influence the Poggendorff illusion in a similar way. To test the effects of these differences, the authors changed the binocular disparity information of the oblique lines and the central rectangle. Specifically, the oblique lines and the central rectangle were presented at different depth planes using liquid-crystal shutter glasses. The results revealed that the strength of the Poggendorff illusion increased when the oblique lines were stereoscopically presented behind but not in front of the central rectangle. Although Wang and Idesawa’s findings corroborate those obtained by Talasli and Inan’s [3], other studies report that increasing the physical width of the central rectangle results in an increase rather than a decrease in the strength of the illusion [5,6,7], indicating contradictory evidence. Therefore, there seems to be no agreement on the effects of adding pictorial depth cues to the Poggendorff illusion and how this might relate to depth processing and perceptual constancy.

Gregory proposed that many other illusions can be explained in a manner similar as he explains the Poggendorff illusion. One such illusion is the Ponzo illusion in which we perceive a stimulus placed at a location where the contextual converging lines indicate greater depth than a stimulus of equal size placed at a location where the contextual converging lines signal less depth. For the Ponzo illusion, Gregory [2,8,9,10] proposed that the brain extracts depth information from the 2D display based on knowing, from previous experience, that linear lines recede into depth. This, in turn, perceptually rescales the size of objects in the image display. Previously, Gillam [11] tested the strength of the Poggendorff illusion using a central area with converging contextual lines, which might be interpreted in the same way as the converging contextual lines of the Ponzo illusion. Her results demonstrated that the presence of a Ponzo-like central area affected the strength of the Poggendorff illusion and concluded that “… the Ponzo illusion and the Poggendorff illusion are attributable to similar processes and are not different kinds of illusions”. Expanding on Gregory’s misapplied perceptual constancy theory, several studies have shown that the strength of many Ponzo-like illusions increases with the number of pictorial depth cues added to the image display [12,13,14,15,16,17,18,19,20]. For example, in a series of recent studies, we added linear perspective cues and textures to a corridor background image and measured the strength of a resulting Ponzo-like illusion [17,18,19,20]. Our results revealed that illusion strength increases with pictorial depth cues added to the image display. In the present investigation, we incorporated a similar paradigm to the Poggendorff illusion and tested the effects of adding pictorial depth cues on the strength of the illusion (Figure 1 and Figure 2). We conducted three experiments using the Method of Constant Stimuli [21].

In the first two experiments, we tested the effects of adding textures (namely, large and small brick patterns) to the central rectangle on the perceived alignment of the oblique lines (experiment 1) and the perceived width of the central rectangle (experiment 2). In both experiments, we presented a central rectangle composed of large bricks (Figure 1B), composed of small bricks (Figure 1C), or consisting of a uniform colour (Figure 1A). We also included a baseline condition without a central rectangle (Figure 1D). We hypothesised that the central rectangle with large bricks, relative to the one with small bricks, would be perceived as being more narrow and would also produce a greater perceptual misalignment of the oblique lines. We also hypothesised a relationship between illusion strength and the perceived width of the central rectangle.

In experiment 3, we tested if adding pictorial depth cues to both the central rectangle and the background image display would affect the Poggendorff illusion. We presented a central rectangle composed of bricks in a corridor rich in texture and linear perspective depth cues (Figure 2A,B), a central rectangle composed of bricks on a uniform background (Figure 2C,D) and a central rectangle of a uniform colour on a uniform background (Figure 2E,F). The central rectangle was presented either in large (Figure 2A,C,E) or small (Figure 2B,D,F) sizes, corresponding to positions in the front and back of the corridor background, respectively. We hypothesised that the addition of pictorial cues would affect the Poggendorff illusion such that the illusion would be stronger when the cues signal that the central rectangle is close to the participant compared to when it is further away.

## 2. Materials and Methods

### 2.1. Participants 

All experiments were conducted during the Coronavirus Disease 2019 (COVID-19) pandemic. We collected data between June and August 2020 for experiments 1 and 2. For experiment 3, we collected data in June 2021. Due to imposed restrictions, all experiments were done entirely online.

Thirty-two participants took part in both experiments 1 and 2 (*M*_Age_ = 24.25 years, *SD* = 3.06, 13 males). Eight participants were removed from the data analysis for not meeting the quality control criteria. We followed a two-step procedure to test whether a participant met the quality control requirements. First, we calculated the mean and standard deviation values for each dependent variable for each condition. Participants who scored three standard deviations above or below the mean values in any instance were removed from the sample. Second, we calculated goodness of fit measures when fitting psychometric curves to the data (see below in Section 2.3.). Participants with goodness of fit values smaller than *r* = 0.63 (corresponding to *p* > 0.05) were removed from the sample. In the final sample, there were 24 participants in both experiments (*M*_Age_ = 24.00 years, *SD* = 2.99, 9 males).

Twenty participants took part in experiment 3 (*M*_Age_ = 25.19 years, *SD* = 3.79, 7 males). As in experiments 1 and 2, we followed the same two-step quality control procedure to flag and exclude outliers. No participants were removed from this procedure. In all three experiments, participants reported to have normal or corrected-to-normal vision and none of them reported to have any previous history of psychiatric and neurological disorders. All participants provided informed consent before the experiments. The participants were compensated with electronic gift cards for their time and any inconveniences. The study was approved by the Human Ethics Committee of La Trobe University.

### 2.2. Procedures

#### 2.2.1. Experiment 1

Experiment 1 was conducted to examine the effects of adding textures to the central rectangles on the magnitude of the Poggendorff illusion. Participants were asked to run the experiments on their own computer in a web browser. Unity (Unity Software Inc., San Francisco, CA, USA, 2004) was used to create the stimuli and call JavaScript functions that controlled trial sequences and experimental blocks. An experienced programmer coded the experiment in Unity. Before starting, participants were asked to provide information about the size and the screen resolution of their monitor so we could ensure beforehand that they could perform the experiment on a suitable device, such as a desktop or notebook computer. The use of a smartphone was not permitted. The Unity program collected the same information when the participant started the experiment and automatically adjusted the size of all stimuli such that they would have the same size for everybody. Due to the online nature of the experiments, we could not use a chin rest. To help ensure that the stimuli subtended the same visual angle on the retina across all participants, we asked participants to measure a distance of 57 cm between their eyes and the screen and to try to maintain this viewing distance throughout the experiment. To store the data collected, we used Heroku (Heroku, San Francisco, CA, USA, 2007). All participants performed the experiment during a Zoom (Zoom Video Communications, San Jose, CA, USA) call with the experimenter using share screen so we could help troubleshoot and ensure compliance.

In experiment 1, four different Poggendorff stimuli were used: (1) the large brick central rectangle, (2) the small brick central rectangle, (3) the uniform central rectangle and (4) the no central rectangle condition (Figure 1). The large brick, small brick and uniform central rectangles had a height and width of 5.9 and 2.1 degrees of visual angle, respectively. The central rectangles with large and small bricks were created using a high-resolution, seamless brick wall image as their texture. The central rectangle with small bricks was created from the central rectangle with the large bricks by reducing the brick sizes. A single brick had a height and width of 0.5 and 1.4 degrees of visual angle for the central rectangle with large bricks while it had a height and width of 0.2 and 0.8 degrees of visual angle for the central rectangle with small bricks. The uniform central rectangle was composed of the average colour of the central rectangles with large and small bricks (R: 160, G: 114, B: 88). Finally, the no central rectangle condition was created by removing the central rectangle from the display altogether.

**Figure 3:**
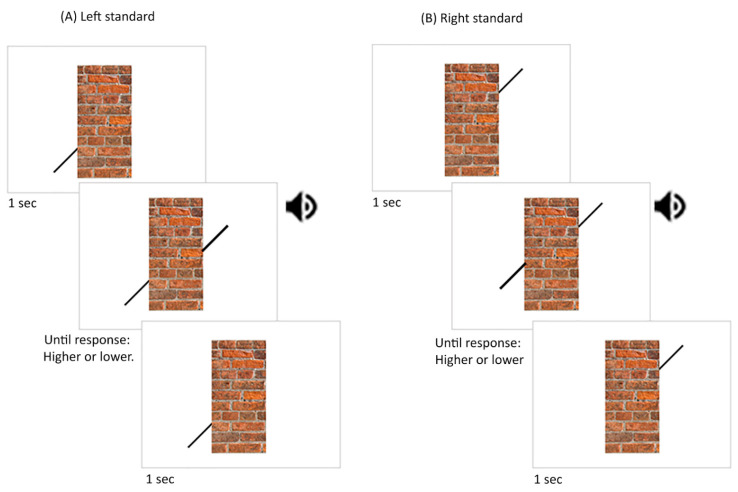
Stimuli and procedures used in experiment 1. For the left standard-line block (**A**), the left standard line was shown for 1 s, followed by an alerting sound cue that signalled the presentation of the right comparison line. For the right standard-line block (**B**), the right standard line was shown for 1 s, followed by an auditory alerting cue that signalled the presentation of the left comparison line. The participant was required to indicate whether the comparison line was higher or lower than the point with which the two lines were colinear. The speaker symbols illustrate when the auditory alerting cues were presented.

There were eight blocks in experiment 1. Each block corresponded to a different condition. In each condition, the perceived alignment of two black (R:0, G:0, B:0) lines was measured using the Method of Constant Stimuli. The lines had a length and width of 1.24 and 0.2 degrees of visual angle, respectively. Both lines were angled 45 degrees from the vertical. One of the lines was designated as the standard stimulus while the other was designated as the comparison stimulus. The standard line was presented on the lower-left part of the Poggendorff stimulus in half of the blocks (e.g., Figure 3A) while it was shown on the upper-right part in the other half of the blocks (e.g., Figure 3B). Therefore, each central rectangle condition was presented twice: once with the standard line on the right and once with the standard line on the left. In the left standard line blocks, the standard line was attached to the left side of the central rectangle 2 degrees of visual angle above the floor line. In the right standard line blocks, the standard line was attached to the right side of the central rectangle 4 degrees above the floor line. The position of the comparison line ranged around the point that was colinear to the standard line in ten 0.3-degree increments. Five increments corresponded to positions higher than the point colinear to the standard line while the five other increments corresponded to the positions lower than that point. There were 100 trials in each block where the comparison line was presented ten times at each position (10 repetitions × 10 increments). The order of blocks and trials within each block was randomised for each participant.

During each block, the standard line was always displayed along with the central rectangle or the gap with the missing central rectangle in the no central rectangle condition. A trial began with a one-second delay (Figure 3). After this delay, an auditory beep was accompanied by the presentation of the comparison line. The comparison line was displayed until participants pressed a button on their keyboard to report whether it was higher or lower than the point that was colinear to the standard line. The comparison line disappeared after the button was pressed. A self-paced break was provided at the end of each block.

#### 2.2.2. Experiment 2

**Figure 4:**
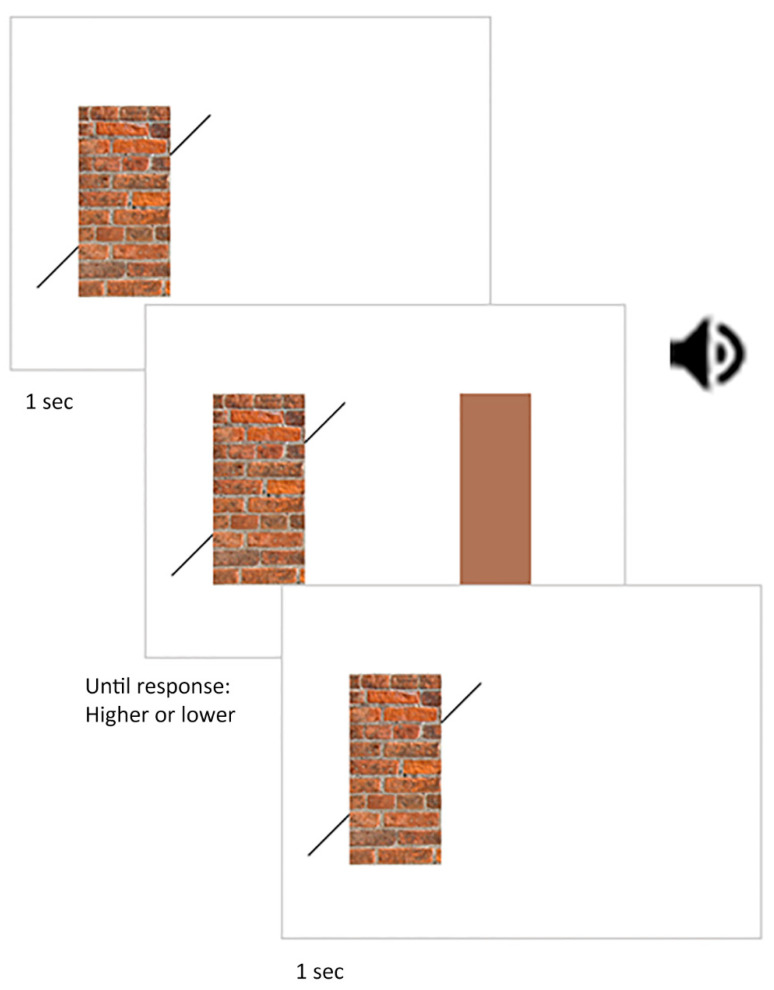
Stimuli and procedures used in experiment 2. In experiment 2, a Poggendorff stimulus with oblique lines was shown for 1 s, followed by an alerting sound cue that signalled the presentation of the comparison rectangle. The participant was required to indicate whether the latter was wider or thinner than the former. The speaker symbol denotes when the auditory alerting cue was presented.

In experiment 2, we measured the perceived width of the same central rectangles used in experiment 1; namely: (1) the central rectangle with large bricks, (2) the central rectangle with small bricks, (3) the uniform central rectangle and (4) the no central rectangle condition (Figure 1). The procedures were similar to those used in experiment 1 except in the following ways. There were four blocks. For each trial, two stimuli were presented side-by-side (Figure 4). The stimulus on the left corresponded to a Poggendorff stimulus and was designated the standard while the one on the right consisted of a rectangle of a uniform colour and was designated the comparison. The Poggendorff stimuli were similar to those used in experiment 1 with the following exceptions. The oblique black lines were always physically colinear and separated by a fixed vertical distance of 2 degrees and a fixed horizontal distance of 2.1 degrees.

The comparison rectangle had a uniform colour corresponding to the average colour of the central rectangles with the large and small bricks (R: 160, G: 114, B: 88). It always had a height of 5.9 degrees of visual angle and its width varied around 2.1 degrees of visual angle in ten 0.1-degree increments. Five increments corresponded to widths larger than 2.1 degrees while the other five were smaller than 2.1 degrees. In each block, there were 100 trials where the comparison rectangle was presented ten times with each width (10 repetitions × 10 increments). The order of blocks and trials within each block was randomised for each participant.

During each block, the Poggendorff stimulus on the left was always displayed. A trial began with a one-second delay (Figure 4). After this delay, an auditory beep was accompanied by the presentation of the comparison rectangle. The comparison rectangle was displayed until participants pressed a button on their keyboard to report whether it was wider or narrower than the central rectangle or the gap corresponding to the missing central rectangle in the Poggendorff stimulus on the left. The comparison rectangle disappeared after the button was pressed. A self-paced break was provided at the end of each block.

#### 2.2.3. Experiment 3

Experiment 3 tested whether adding pictorial depth cues to both the central rectangle and the background image display would affect the Poggendorff illusion. An online experiment was conducted in Gorilla (Cauldron Science, Cambridge, UK, 2009). We decided to change to the Gorilla platform due to its ease of use. As in the other experiments, participants performed the experiment on their own computers in a web browser under Zoom supervision using the screen share option. The use of smartphones was not permitted; only desktops and notebooks were permitted. At the start of the session, the participants were asked to complete a calibration task. In this task, participants placed a credit card on the screen and adjusted the size of a rectangle besides it so that the two matched. Based on the information gathered, the size of the stimuli in the entire experiment was adjusted automatically in Gorilla so that all participants were presented with same-sized stimuli. To help ensure that stimuli subtended the same visual angle on the retina in all participants, we asked them to measure a distance of 57 cm between their eyes and the screen and try to maintain this viewing distance throughout the experiment.

We presented a central rectangle composed of bricks in a corridor rich in texture and linear perspective depth cues (Figure 2A,B), a central rectangle composed of bricks on a uniform background (Figure 2C,D) and a central rectangle of a uniform colour on a uniform background (Figure 2E,F). The central rectangle was presented either in large (Figure 2A,C,E) or small (Figure 2B,D,F) sizes, corresponding to positions in the front and the back, respectively, of the corridor background. The large central rectangle had a height and width of 9.8 and 5.2 degrees of visual angle, respectively (Figure 2A,C,E). The small central rectangle had a height and width of 3.5 and 1.8 degrees of visual angle, respectively (Figure 2B,D,F). The central rectangle with bricks was created using a high-resolution, seamless brick wall image as a texture cue. A single brick had a height and width of 0.5 and 1.4 degrees of visual angle for the central rectangles with large bricks while it had a height and width of 0.1 and 0.3 degrees of visual angle for the central rectangles with small bricks. The colour of the uniform rectangle corresponded to the average colour of the large and small central rectangles (R: 183, G: 132, B: 101).

To render the corridor background, we created a 3D virtual environment of a hallway in Autodesk 3ds Max (Autodesk, Inc., San Rafael, CA, USA) (Figure 2A,B). The floor and ceiling of the hallway were 1100 cm in length and 425 cm in width. The left and right walls were 1100 cm in length and 425 cm in height. The side walls were 425 cm away from each other. A back wall was placed at the end of the hallway. The back wall had a height of 425 cm. All walls were textured with a high-resolution, seamless brick wall image. A virtual camera was placed 1100 cm away from the back wall to take a picture of the virtual environment. The virtual camera was set to a full-frame of 35 mm, a focal length of 29 mm and an aperture of f/8. Global lighting simulated daylight (6500 K). The rendered corridor background with bricks was cropped in Adobe Photoshop. The colour of the background without the corridor corresponded to the average colour of the corridor background (R: 196, G: 142, B: 108).

The perceived alignment of two black (R:0, G:0, B:0) lines was measured using the Method of Constant Stimuli. The lines had a length and width of 1.2 and 0.2 degrees of visual angle, respectively. Both lines were angled 45 degrees from the vertical. The line on the lower-left portion of the Poggendorff figure was designated as the standard while the line on the upper-right portion of the Poggendorff figure was designated as the comparison. Given that we did not find any differences in illusion strength in experiment 1 between blocks where the standard was on the left versus the right side, we did not think it was necessary to test both configurations again in experiment 3.

There were six blocks, each corresponding to one of the six conditions (Figure 2). The standard line was always attached to the central rectangle on the left side 1.8 and 0.4 degrees of visual angle above the floor-line for the large and small central rectangle blocks, respectively. In the large central rectangle blocks, the position of the comparison line ranged around the point that was colinear to the standard line in ten 0.4-degree increments. In the small central rectangle blocks, the position of the comparison line ranged around the point that was colinear to the standard line in ten 0.2-degree increments. In both the large and small central rectangle blocks, five increments corresponded to the positions higher than the point colinear to the standard line while the other five increments corresponded to the positions lower than that point. There were 100 trials in each block where the comparison line was presented ten times at each position (10 repetitions × 10 increments). The order of blocks and trials within each block was randomised for each participant.

The trial sequence was similar to that in experiment 1. Namely, during each block, the standard line was always displayed with the central rectangle. A trial began with a one-second delay (Figure 3A). After this delay, an auditory beep was accompanied by the presentation of the comparison line. The comparison line was displayed until participants pressed a button on their keyboard to report whether it was higher or lower than the point that was colinear to the standard line. The comparison line disappeared after the button was pressed. In contrast to experiments 1 and 2, reaction times were measured in this experiment to denote the time it took participants to press a button after the comparison line appeared. A self-paced break was provided at the end of each block.

### 2.3. Statistical Analyses

We created psychometric curves for each condition for each participant. In experiments 1 and 3, the number of instances in which the participant reported the comparison line position as appearing higher than the point colinear to the standard line was tabulated for each increment. In experiment 2, the number of instances in which the participant reported the width of the comparison rectangle as appearing wider than the standard (i.e., the central rectangles or the gap of the missing central rectangle under the no central rectangle condition) was tabulated for each increment. Then, the following logistic function was used to calculate the probability (*P*) of the participant reporting the comparison line position as appearing higher in experiments 1 and 3 and the comparison rectangle as appearing wider in experiment 2.

(1) $$P\left(x\right)=\frac{e^{b0+b1x}}{1+e^{b0+b1x}}$$

The estimated probabilities were based on a general linear model fit (*b*0 and *b*1 denote coefficient estimates). The point of subjective equality (PSE) was calculated as *P* = 0.5 from this function. In experiments 1 and 3, the PSE represented how high the comparison line needed to be for the participant to consider this line as appearing at the point that was colinear to the standard line. In experiment 2, the PSE represented how wide the comparison rectangle needed to be to match the width of the standard. Eight, four and six psychometric curves were created for each participant in experiments 1, 2 and 3, respectively. The resulting curves of the model fit well across the conditions for each participant (*r* ranging between 0.659 to 0.997 in experiment 1, 0.800 to 0.997 in experiment 2 and 0.654 and 0.997 in experiment 3). Additionally, we calculated the bistability width (ω) of each PSE curve as follows:

(2) $$\omega =P_{0.75}-P_{0.25}$$

In the above function, *P*_0.25_ corresponds to the result of the logistic function for x when *P* = 0.25. Similarly, *P*_0.75_ corresponds to the result of the logistic function for x when *P* = 0.75. The bistability width was used to estimate the degree of uncertainty in each participant’s responses. Higher bistability width values were considered as signifying a greater perceptual uncertainty. The resulting PSE values and bistability widths were analysed using null-hypothesis statistical testing (NHST). Statistical analyses were carried out using JASP software version 0.11.1.0 (University of Amsterdam, Amsterdam, The Netherlands). All reported *p* values were corrected for multiple comparisons unless specified otherwise. We applied Greenhouse–Geisser corrections whenever the assumption of sphericity was not met in Mauchly’s sphericity test. Post-hoc pairwise comparisons were performed to examine effects and interactions found significant by analyses of variance (ANOVA). We used the Bonferroni method to correct for multiple comparisons when making these pairwise comparisons. Additional tests, specific to each experiment, are explained next and in the Section 3, as needed.

#### 2.3.1. Experiment 1

One-sample *t*-tests were conducted to determine if the line that was colinear to the standard appeared different from its physical position. We used the Bonferroni method to correct eight one-sample *t*-tests. We also performed a 2 × 4 ANOVA with the visual field ((1) left standard line, (2) right standard line) and Poggendorff stimulus ((1) large brick central rectangle, (2) small brick central rectangle, (3) uniform central rectangle, (4) no central rectangle) as within-subject factors. Before entering PSEs into the ANOVA, we multiplied −1 to values obtained when the standard was on the left such that we could compare absolute magnitudes between when the standard was on the left versus the right.

#### 2.3.2. Experiment 2

One-sample *t*-tests were conducted to determine if the standard appeared different from its physical width (2.1 cm). We used the Bonferroni method to correct four one-sample *t*-tests. We also performed one-way ANOVA with Poggendorff stimulus ((1) large brick central rectangle, (2) small brick central rectangle, (3) uniform central rectangle, (4) no central rectangle) as a within-subject factor.

#### 2.3.3. Experiment 3

One sample *t*-tests were conducted to test if the comparison line in each condition was perceived differently than the line that was physically colinear to the standard. We used the Bonferroni method to correct six one-sample *t*-tests. We also performed a 2 × 3 ANOVA with the central rectangle size ((1) large central rectangle, (2) small central rectangle) and Poggendorff stimulus ((1) corridor background with brick central rectangles, (2) uniform background with brick central rectangles, (3) uniform background and uniform central rectangles) as within-subject factors. We repeated the above ANOVA on reaction time data.

## 3. Results

### 3.1. Experiment 1

Experiment 1 was conducted to examine the effects of adding pictorial depth cues to the central rectangle on the magnitude of the Poggendorff illusion. In short, the results demonstrate that the presence of a central rectangle, regardless of its textural composition, determined the strength of the illusion. There were no differences in bistability widths between conditions.

**Figure 5:**
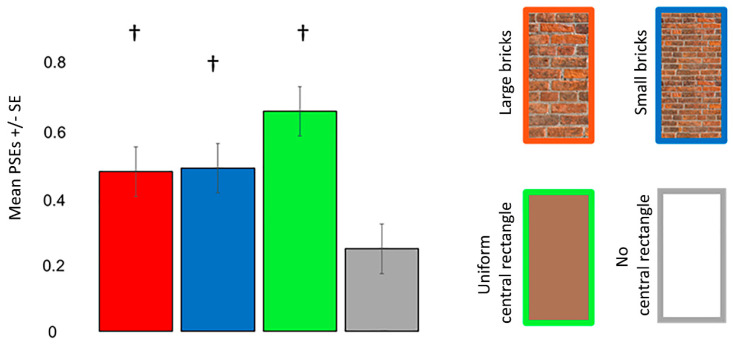
PSEs for the different Poggendorff stimuli in experiment 1. Daggers (†) denote differences from the no central rectangle condition at *p* < 0.050 after correcting for multiple comparisons using the Bonferroni method. PSEs were computed from psychometric functions. PSEs in left standard line blocks were multiplied by −1 before the analysis. Error bars denote standard errors for within-subject contrasts as described by O’Brien and Cousineau [22].

One sample *t*-tests revealed that the participants perceived a misalignment of the oblique lines in each condition when the standard line was presented on the left (all *p_corr_* < 0.001) and right (all *p_corr_* ≤ 0.022) sides. ANOVA revealed that the main effect of the Poggendorff stimulus (*F* (2, 29) = 8.197, *p* = 0.005, $\eta_{p}^{2}$ = 0.263, Greenhouse–Geisser corrected) but not the visual field (*F* (1, 23) = 0.209, *p* = 0.652, $\eta_{p}^{2}$ = 0.009) was significant. The interaction between the visual field and Poggendorff stimulus was not significant (*F* (2, 29) = 1.274, *p* = 0.278, $\eta_{p}^{2}$ = 0.053, Greenhouse–Geisser corrected). Post-hoc comparison tests carried out to further examine the main effect of the Poggendorff stimulus demonstrated that the no central rectangle condition produced a weaker illusion than the large brick central rectangle (*t* = 2.767, *p_corr_* = 0.044, *d* = 0.565), the small brick central rectangle (*t* = 2.892, *p_corr_* = 0.031, *d* = 0.590) and the uniform central rectangle (*t* = 4.931, *p_corr_* < 0.001, *d* = 1.007) (Figure 5). There were no differences between the large and small brick central rectangles (*t* = 0.124, *p_corr_* > 0.999, *d* = 0.025) nor were there any differences between the uniform central rectangle versus the large (*t* = 2.164, *p_corr_* = 0.204, *d* = 0.442) and small (*t* = 2.039, *p_corr_* = 0.272, *d* = 0.416) brick central rectangles. 

**Figure 6:**
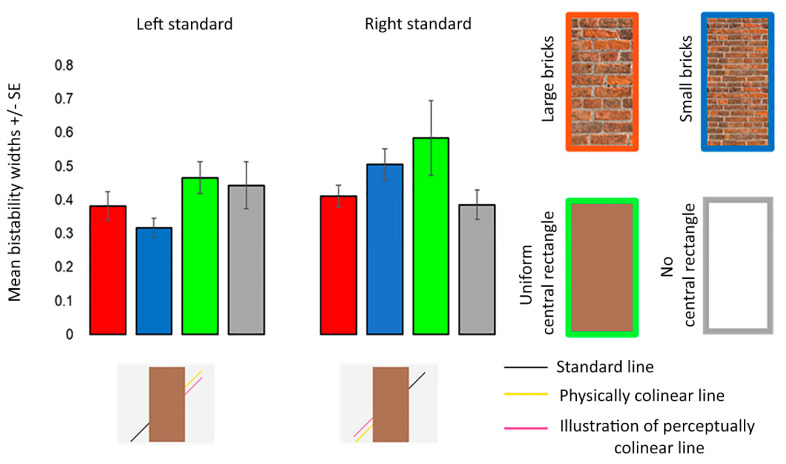
Mean bistability widths for the different Poggendorff stimuli in experiment 1. Mean bistability widths were computed by subtracting *P* = 0.25 from *P* = 0.75. Error bars denote standard errors for within-subject contrasts as described by O’Brien and Cousineau [22].

For bistability widths, ANOVA revealed that the main effects of the Poggendorff stimulus (*F* (3, 52) = 2.370, *p* = 0.097, $\eta_{p}^{2}$ = 0.093, Greenhouse–Geisser corrected) and visual field (*F* (1, 23) = 2.050, *p* = 0.166, $\eta_{p}^{2}$ = 0.082) were insignificant. The interaction between these two factors did not reach significance (*F* (2, 39) = 1.696, *p* = 0.200, $\eta_{p}^{2}$ = 0.069, Greenhouse–Geisser corrected) (Figure 6).

### 3.2. Experiment 2

In experiment 2, we measured the perceived width of the same central rectangles used in experiment 1. In short, there was some evidence of perceptual size rescaling. The small brick central rectangle was perceived as wider than the uniform central rectangle, and the large brick central rectangle was perceived as narrower than the gap where there was no central rectangle in the no central rectangle condition. Bistability widths decreased for the large brick central rectangle.

**Figure 7:**
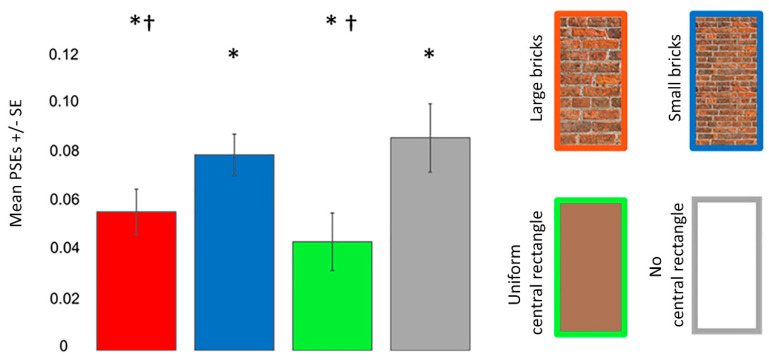
PSEs for the different Poggendorff stimuli in experiment 2. Daggers (†) denote differences from the no central rectangle condition at *p* < 0.050 after correcting for multiple comparisons using the Bonferroni method. Asterisks (*) denote differences from the physical width at *p* < 0.050 after correcting for multiple comparisons using the Bonferroni method. PSEs were computed from psychometric functions. Error bars denote standard errors for within-subject contrasts as described by O’Brien and Cousineau [22].

Figure 7 shows the mean PSEs with error bars for each Poggendorff stimulus. One sample *t*-tests revealed that each Poggendorff stimulus was perceived wider than its physical width (all *p_corr_* ≤ 0.001). ANOVA revealed that the main effect of the Poggendorff stimulus (*F* (3, 69) = 6.431, *p* < 0.001, $\eta_{p}^{2}$ = 0.219) was significant. Post hoc comparisons demonstrated that the no central rectangle condition was perceived to be wider than both the large brick (*t* = 2.739, *p_corr_* = 0.047, *d* = 0.559) and uniform (*t* = 3.837, *p_corr_* = 0.002, *d* = 0.783) central rectangles. The difference between the perceived widths of the large and small brick central rectangles was insignificant (*t* = 2.112, *p_corr_* = 0.230, *d* = 0.431). The small brick central rectangle was perceived to be wider than the uniform central rectangle (*t* = 3.210, *p_corr_* = 0.012, *d* = 0.655).

**Figure 8:**
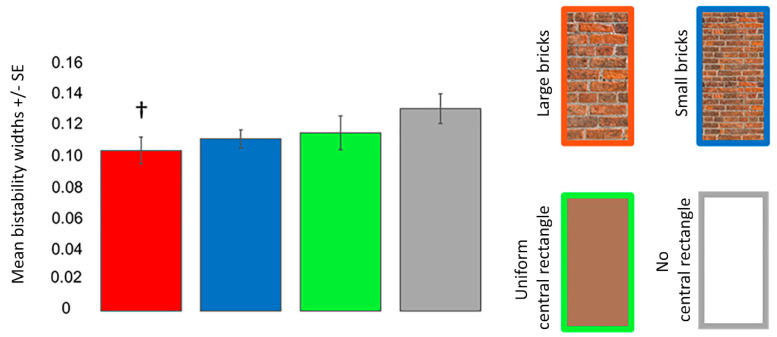
Mean bistability widths for the different Poggendorff stimuli in experiment 2. The dagger (†) denotes a difference from the no central rectangle condition at *p* < 0.050 after correcting for multiple comparisons using the Bonferroni method. Mean bistability widths were computed by subtracting *P* = 0.25 from *P* = 0.75. Error bars denote standard errors for within-subject contrasts as described by O’Brien and Cousineau [22].

For bistability widths, ANOVA revealed that the main effect of the Poggendorff stimulus (*F* (2, 54) = 3.254, *p* = 0.039, $\eta_{p}^{2}$ = 0.124, Greenhouse–Geisser corrected) was significant (Figure 8). Post hoc comparison tests demonstrated that the large brick central rectangle resulted in less perceptual uncertainty than the no central rectangle condition (*t* = 3.025, *p_corr_* = 0.021, *d* = 0.618). There were no other significant differences (all *p_corr_* ≥ 0.192).

### 3.3. Correlations between Illusion Strength and Perceived Width

Taken together, the results so far suggest that changes in the perceived width of the central rectangle did not affect the strength of the Poggendorff illusion if one considers that differences in perceived width between the conditions in experiment 2 did not parallel differences in illusion strength in experiment 1. To confirm this, we further examined if the perceived misalignment of the oblique lines, as measured in experiment 1, correlated with the perceived width of each Poggendorff stimulus, as measured in experiment 2. We could perform these correlations because the same participants participated in the two experiments. The resulting Pearson correlation coefficient values (*r*) were insignificant (large brick central rectangle: *r* (22) = 0.249, *p_uncorr_* = 0.240; small brick central rectangle: *r* (22) = 0.250, *p_uncorr_* = 0.239; uniform central rectangle: *r* (22) = 0.298, *p_uncorr_* = 0.158; no central rectangle condition: *r* (22) = −0.094, *p_uncorr_* = 0.661).

### 3.4. Experiment 3

In experiment 3, we tested whether adding pictorial depth cues to both the central rectangle and the background image display would affect the Poggendorff illusion. To summarise the main findings, the illusion was strongest and demonstrated greater perceptual uncertainty when the central rectangle was large as opposed to small, regardless of the addition of pictorial depth cues to the central rectangle or the background. Moreover, the uniform central rectangle on the uniform background produced a stronger illusion than the brick central rectangle on both the corridor and uniform backgrounds.

**Figure 9:**
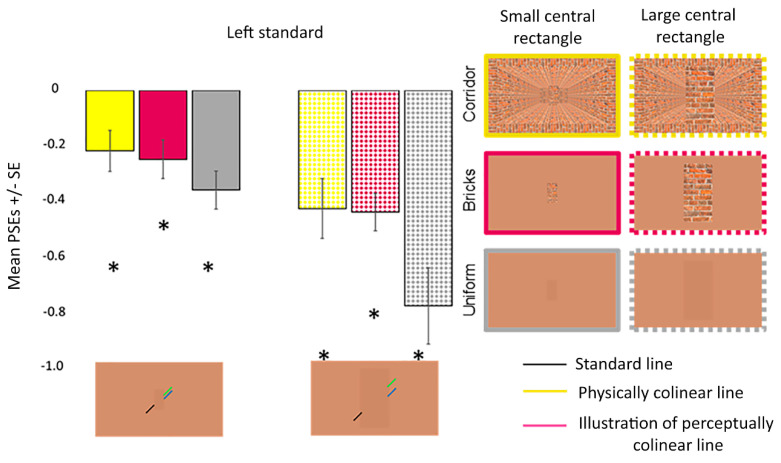
PSEs for the different conditions in experiment 3. Asterisks (*) denote differences from the physical width at *p* < 0.050 after correcting for multiple comparisons using the Bonferroni method. PSEs were computed from psychometric functions. Error bars denote standard errors for within-subject contrasts as described by O’Brien and Cousineau [22].

Figure 9 shows the mean PSEs with error bars for the large and small central rectangles for each background. One sample *t*-tests results revealed that the participants perceived misalignment in each condition (*p_corr_* ≤ 0.012) (Figure 9). ANOVA revealed that the interaction between the central rectangle size and Poggendorff stimulus did not reach significance (*F* (2, 38) = 2.837, *p* = 0.071, $\eta_{p}^{2}$ = 0.130). On the other hand, there were main effects of the central rectangle size (*F* (1, 19) = 11.364, *p* = 0.003, $\eta_{p}^{2}$ = 0.374) and Poggendorff stimulus (*F* (2, 38) = 13.495, *p* < 0.001, $\eta_{p}^{2}$ = 0.415) (Figure 9). Large central rectangles (*M* = 0.54) produced a greater perceptual misalignment than the small central rectangles (*M* = 0.27). Post-hoc comparisons demonstrated that the uniform central rectangle on the uniform background produced a stronger illusion than the brick central rectangle on both the corridor (*t* = 4.319, *p_corr_* < 0.001, *d* = 0.966) and uniform (*t* = 4.333, *p_corr_* < 0.001, *d* = 0.969) backgrounds. Illusion strength did not differ when the brick central rectangle was presented on the corridor versus uniform backgrounds (*t* = 0.444, *p_corr_* > 0.999, *d* = 0.969).

**Figure 10:**
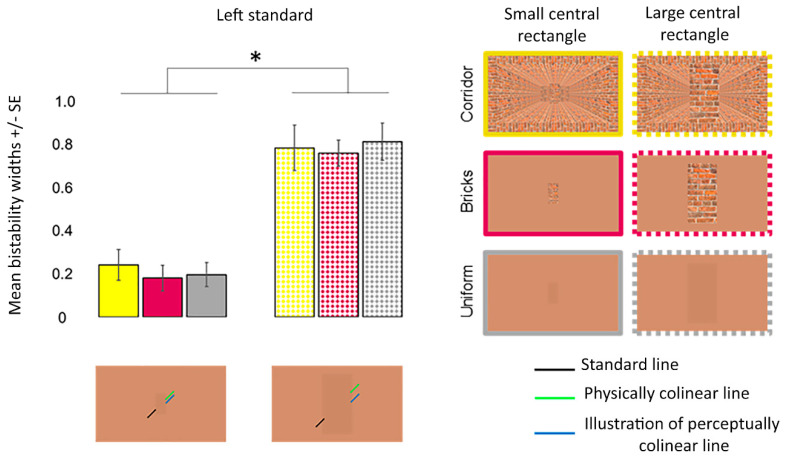
Mean bistability widths for the different conditions in experiment 3. The asterisk (*) denotes the difference between the perceptual uncertainty produced by the large and small central rectangles at *p* < 0.050. Mean bistability widths were computed by subtracting *P* = 0.25 from *P* = 0.75. Error bars denote standard errors for within-subject contrasts as described by O’Brien and Cousineau [22].

For bistability widths, ANOVA revealed that the main effect of the central rectangle size (*F* (1, 19) = 143.608, *p* < 0.001, $\eta_{p}^{2}$ = 0.883) but not the Poggendorff stimulus (*F* (1, 26) = 0.395, *p* = 0.597, $\eta_{p}^{2}$ = 0.020) was significant (Figure 10). The interaction between the two factors did not reach significance (*F* (1, 26) = 0.257, *p* = 0.689, $\eta_{p}^{2}$ = 0.013). The main effect of the central rectangle size was driven by greater perceptual uncertainty for the large (*M* = 0.79) compared to the small (*M* = 0.21) central rectangle.

For reaction times, ANOVA revealed that the main effect of the central rectangle size (*F* (1, 19) = 0.427, *p* = 0.521, $\eta_{p}^{2}$ = 0.022) was not significant. The main effect of the Poggendorff stimulus (*F* (2, 23) = 3.773, *p* = 0.059, $\eta_{p}^{2}$ = 0.166, Greenhouse–Geisser corrected) trended towards significance. Namely, reaction times tended to be slower when the central rectangle was presented in the corridor (*M* = 782.96 milliseconds) versus the uniform backgrounds (the uniform central rectangle on the uniform background: *M* = 736.54 milliseconds; the brick central rectangle on the uniform background: *M* = 710.23 milliseconds). The interaction between the two factors did not reach significance (*F* (2, 38) = 0.936, *p* = 0.401, $\eta_{p}^{2}$ = 0.047).

## 4. Discussion

The present investigation examined the effects of adding pictorial depth cues to the Poggendorff illusion. For experiments 1 and 2, we reasoned that if the illusion is driven by perceptual size rescaling mechanisms, then the central rectangle with large bricks, relative to the one with small bricks, would be perceived to be more narrow and would also produce a greater perceptual misalignment of the oblique lines. More broadly, we expected that the illusion strength would increase under conditions where participants perceived a narrower central rectangle. Our results did not reveal any differences in illusion strength between the central rectangles with large and small bricks, nor did they indicate differences between their perceived widths. Although there was evidence of perceptual size rescaling whereby the central rectangle with large bricks was perceived as narrower than the no central rectangle condition and the central rectangle with small bricks was perceived as wider than the uniform central rectangle, these changes did not affect the strength of the illusion. Further, illusion strength in experiment 1 did not correlate with the perceived width of the central rectangle in experiment 2.

In experiment 3, we tested whether adding pictorial depth cues to both the central rectangles and the background image display would affect the Poggendorff illusion. Contrary to our hypothesis, our results revealed that adding pictorial depth cues to both the central rectangles and the background decreased rather than increased the strength of the illusion. Illusion strength also changed depending on the size of the central rectangle. The large central rectangle produced a stronger illusion than the small central rectangle. Overall, the results of the three experiments suggest that the illusion is not driven by perceptual rescaling mechanisms that shrink the apparent width of the central rectangle. On the other hand, adding pictorial depth cues seems to decrease the strength of the illusion, which is what Gillam [11], as we will discuss in more detail later, predicted based on her reasoning that adding pictorial depth cues increases rather than decreases the certainty of the linear perspective created by the oblique lines. The following discussion focuses on how the results might relate to low-level and more ‘cognitive’ based theories to explain the illusion and argue that it could be the combination of both given our results. Throughout the discussion, we use the term ‘cognitive’ loosely to denote explanations that are not predominantly based on bottom-up processes fulfilled by retinal, thalamic and early visual areas and that place more importance on other processes mediated elsewhere in the brain or their top-down influences.

### 4.1. Empirical Arguments for and against Theories That Explain the Poggendorff Illusion with Mechanisms Related to Depth Perception

Talasli and Inan [3] explained the perceived misalignment in the Poggendorff illusion by mechanisms that play a role in extracting depth information from 2D images. The authors proposed that the central rectangle is treated as an occluder and that its supposed nearness causes an illusory misalignment in the oblique lines by inappropriately triggering perceptual size rescaling mechanisms. Namely, the central rectangle acting as an occluder is treated as being more narrow than it really is, consequently causing a supposed shift in the positioning of the oblique lines towards the midline and making them appear more misaligned. Our findings in experiment 3 demonstrating a stronger illusion for uniform central rectangles compared to central rectangles with bricks contradicts the Talasli and Inan [3] study. However, our findings agree more with a different study showing that the presence of dot patterns can cause a decline in the strength of the Poggendorff illusion [23]. Masini et al. [23] examined how the magnitude of the Poggendorff illusion changes with variations in the textural composition of the central rectangle. They demonstrated that the strength of the Poggendorff illusion was greater when the central rectangle was either empty or completely filled with dot patterns. Based on these results, the authors suggested that the Poggendorff illusion’s strength decreases when there are dot patterns with gaps because these patterns can cause a perceptual enlargement in the width of the central rectangle. In line with this idea, our results revealed that the central rectangle with small bricks was perceived to be wider than the uniform one in experiment 2.

Gillam [11] also postulated that learned processes related to the extraction of depth play an important role in the Poggendorff illusion but in a manner that differed from the one proposed by Talasli and Inan [3] and that originally formulated the hypotheses for our study. Gillam [11] did not predict an increase in the strength of the illusion with the perceived shrinkage of the central rectangle nor did she predict that adding pictorial depth cues like we did would increase its strength. Rather, Gillam [11] proposed that the illusion is driven primarily by the brain treating the two oblique lines as different lines receding into depth, which in turn causes a perceptual distortion in their alignment. According to her account, known as depth processing theory, the addition of other pictorial depth cues in the background adds greater certainty to whether the oblique lines can be considered to be one line receding into the distance, causing a decline in the magnitude of their perceived misalignment.

**Figure 11:**
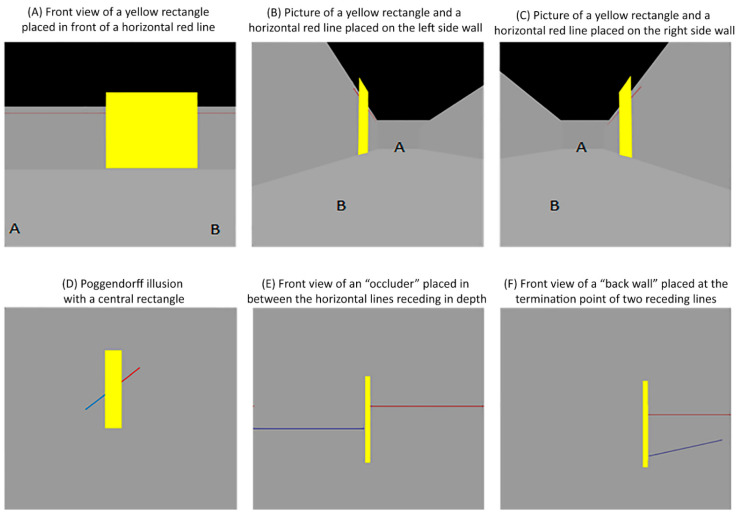
Poggendorff figures used to illustrate the possible interpretations of attached lines and central areas. Pictures of the virtual environments were taken using a virtual camera in Autodesk 3dsMax. (**A**) Front view of a yellow rectangle placed in front of a horizontal red line. The front view image of the yellow rectangle was taken by placing a virtual camera in between points A and B (see Video S1). (**B**) Side view of a yellow rectangle placed in front of a horizontal red line on the left side wall. (**C**) Side view of a yellow rectangle placed in front of a horizontal red line on the right side wall (see Video S1). (**D**) Poggendorff illusion with a central rectangle (see Video S2). (**E**) Front view of an “occluder” placed in between the horizontal lines receding in depth. The side view of this image was illustrated in Figure 2D (see Video S3). (**F**) Front view of a “back-wall” placed at the termination point of two lines receding in depth. The side view of this image was illustrated in Figure 2D (see Video S4).

To aid clarity, imagine you are walking from point A to B inside a corridor. You see a yellow rectangle placed in front of a horizontal red line on one of the sidewalls (see Figure 11A and Video S1). If you take pictures of the yellow rectangle from the end of the corridor (e.g., point B in Figure 11B,C, also see Video S1), the rectangle and the line on the left sidewall would appear as in Figure 11B, while the rectangle and the line on the right sidewall would appear as in Figure 11C. Gillam [11] proposed that when the parts of the central area are rescaled in size, as in Figure 11B,C, the visual system processes the lines attached to the central area as if they are colinear horizontal lines receding into the distance. Thus, the perceptual rescaling of the central area serves as a pictorial depth cue, and this pictorial depth cue adds greater certainty as to whether the oblique lines can be considered to be one line receding into the distance, consequently causing a decline in the magnitude of their perceived misalignment. Contrarily, when the parts of the central area are not rescaled in size, as in the yellow central rectangle in Figure 11D, the visual system does not process the oblique lines as a single horizontal line as in Video S2. Rather, the visual system processes the points attached to the central rectangle as if they were placed at the same distance from the viewer in the 3D environment (see Videos S3 and S4).

Gillam [11] argued that when the attached points are at the same distance, this 2D representation could only result if the receding right line was placed at a lower position than the left one. Importantly, multiple lines and central rectangle positions might result in the same 2D representation (see Figure 11E,F and Videos S3 ans S4) [6,7,24,25]. Namely, the central rectangle can be interpreted as either an occluder placed in between the horizontal lines receding in depth (Figure 11E, Video S3) or a back wall placed at the termination point of two receding lines (Figure 11F, Video S4). As the physical properties of 3D objects are conflated in their 2D representations [6,7,24], one may even argue that the central rectangle might be interpreted as a 2D representation of a 3D trapezoid with a shorter base placed at a nearer distance. Gillam’s [11] theory asserts that it is this uncertainty about the position of oblique lines receding in the distance that creates an apparent misalignment in the oblique lines. In other words, when the central area is not perceptually rescaled, the absence of this pictorial depth cue creates an uncertainty about the positions of the receding lines in 3D space, which in turn causes an increase in the magnitude of their perceived misalignment.

**Figure 12:**
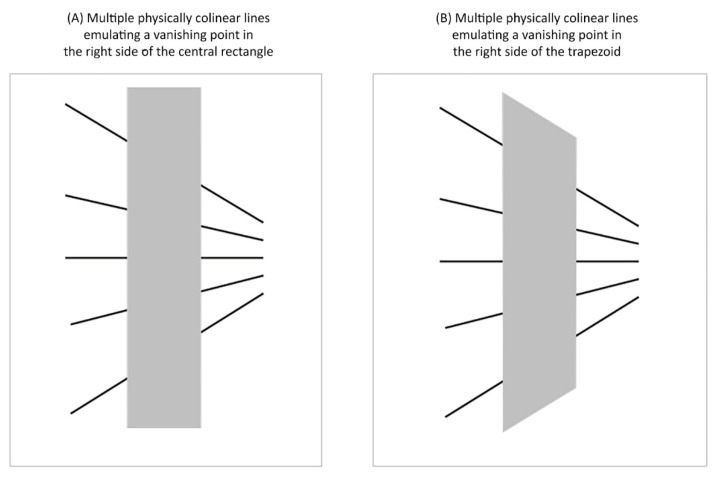
Poggendorff figures used to illustrate the possible effects of pictorial depth cues on the strength of the Poggendorff illusion. (**A**) Multiple physically colinear lines emulate a vanishing point on the right side of the rectangular central rectangle. (**B**) Multiple physically colinear lines emulate a vanishing point on the right side of the central trapezoid-like shape. Similar central areas were used by Gillam [11] to illustrate the effects of pictorial depth cues on the strength of the Poggendorff illusion. Gillam [11] demonstrated that the strength of the Poggendorff misalignment was weaker in Panel B and suggested that this was because it could be interpreted as a left-sidewall of a corridor.

Similar to these ideas, Gillam [11] demonstrated that the strength of a typical Poggendorff illusion in Figure 12A is stronger than the variant in Figure 12B. She argued that this was due to a decrease in the certainty of the oblique lines representing continuous lines receding in the distance in the former case. Thus, according to Gillam, one way to diminish uncertainty about collinearity would be to increase the number of pictorial depth cues in the scene. In line with her predictions, we demonstrated that the strength of the Poggendorff illusion decreased as pictorial depth cues were added to the image display.

Many theories on the Poggendorff illusion do not consider depth processing and size constancy but instead ascribe a greater role to low-level mechanisms. For example, some have proposed that neural circuits in early visual areas related to the processing of acute and obtuse angles create the Poggendorff illusion [26,27,28,29]. Previously, Day and Dickinson [30] demonstrated that the perceived misalignment of the oblique lines in the Poggendorff illusion could be driven by a combination of three different visual illusions driven by low-level mechanisms–namely, the horizontal–vertical, obtuse–acute angles and longitudinal–transversal illusions. The authors posited that mechanisms underlying the horizontal–vertical illusion operate so that the participants underestimate the horizontal distance between the two oblique lines while overestimating the vertical distance between them, similar to how Talasli and Inan [3] explained how changes in the perceived width of the central rectangle could create a change in the apparent alignment of the oblique lines. In line with this explanation, Gregory [2] proposed that the horizontal–vertical illusion is a simple visual illusion that is found in and drives many more complicated illusions.

With this in mind, there could be an alternative explanation as to why adding pictorial depth cues in the background might have decreased the illusion strength in experiment 3. Perhaps perceptual rescaling mechanisms drive the horizontal–vertical component of the Poggendorff illusion while low-level mechanisms drive the obtuse–acute angle component of the illusion. Our experiments provide evidence against the role of the horizontal–vertical component of the Poggendorff illusion by revealing that neither a physically nor perceptually narrower central rectangle produces greater misalignment.

Our findings cannot provide direct evidence for or against low-level explanations because our experiments were not designed to test them. Yet, some of our findings do corroborate these theories to some degree. For example, in experiment 3, we showed that the strength of the illusion varied as a function of the physical width of the central rectangle. Since effects of obtuse and acute angles on the strength of the Poggendorff illusion increase with an increase in the width of the central rectangle [30], this finding can be seen as evidence for theories that explain the Poggendorff illusion with low-level mechanisms that play a role in processing obtuse and acute angles by early visual areas.

Yet, studies showing Poggendorff illusions during sequential [31], dichoptic [32] and illusory contour [33] presentations have challenged these low-level explanations. For example, Sugita et al. [31] investigated the temporal and spatial integration of the illusion by presenting the oblique lines and the central rectangle briefly (16.7 ms) sequentially in different orders (i.e., lines first versus occluder first) with various interstimulus intervals. The results showed that temporally separating the central rectangle 50 ms before to 200 ms after the presentation of the oblique lines induced a significant illusion with especially strong effects when the central rectangle was presented 100 to 150 ms after the oblique lines. Based on these findings, the authors concluded that the initial representation of oblique lines in iconic memory was altered by subsequent processing of the central rectangle, suggesting that the illusion might not be confined to low-level mechanisms.

Along the same vein, in an fMRI study, Shen et al. [33] demonstrated that a Poggendorff illusion can occur with the creation of an apparent central area from Kanizsa-like illusory stimuli. The authors further reported common neural activation patterns for the Poggendorff illusion produced from physical and illusory contours, particularly in areas of the parietal lobe. Interestingly, we also report a significant Poggendorff illusion arising from the no central rectangle condition in experiment 1. It could be the case that these effects were driven by imagery. Taken together, these observations suggest once again that the illusion might not be confined to low-level mechanisms.

### 4.2. Methodological Considerations

One could argue that the effects induced by our central rectangles with small and large brick patterns on how the brain treats distance may have not been strong enough to cause a perceptual distortion in their widths. Talasli and Inan [3] asked their participants to report the apparent distance of their central rectangles. In total, 27 of their 34 participants reported the central rectangle with the small bricks to appear more distant than the one with large bricks. We did not perform this test and remain sceptical about the validity of the perceived distances reported by Talasli and Inan [3] because people are consciously aware that 2D images have no depth. It is more likely that these reports were influenced by response biases [34].

Gregory [9,35,36] acknowledged this problem years ago by noting the paradoxical nature of 2D images with pictorial depth cues. According to him, perceptual rescaling mechanisms operate so that the brain extracts depth information from 2D representations of 3D scenes automatically. This automaticity causes the visual system to still process depth information arising from pictorial cues despite people’s conscious awareness that pictures have no real depth and the conflicting extra-retinal cues, such as vergence and accommodation, telling the brain that pictures are flat. To overcome this problem and properly test for perceived distance differences induced by pictorial depth cues without these other influences, Gregory [35] constructed a device called a Pandora’s box that made use of mirrors and polarising lights to create illusion stimuli viewed monocularly that appeared to subtend in space.

Several studies have used this device to examine how the brain might treat pictorial input as depth cues in geometrical illusions. For example, Chevrier and Delorme [13] examined the relationship between the strength of a Ponzo-like illusion and perceived depth. Their results revealed increased perceptual distortions in stimulus size when participants perceived a greater distance between stimuli placed at locations where pictorial depth cues signal little depth and stimuli placed at locations where pictorial depth cues signal greater depth. Likewise, future work could examine the relationship between perceived distance and the strength of the Poggendorff illusion using a Pandora’s box. Alternatively, the same principles as a Pandora’s box can be applied in a virtual reality experiment to remove all depth cues other than those that are part of the illusion stimulus.

Another consideration is whether the effects of textures in experiment 3 were driven by differences in spatial frequency compositions rather than differences in how they might represent depth. Since textures are defined by spatial frequencies, it is impossible to disentangle their effects at a sensory level. Nonetheless, given that we found no difference between the strength of the Poggendorff illusion produced by the central rectangles with small and large bricks in experiment 1, it is more likely the case that the effects of adding bricks in experiment 3 were driven by their conceptual processing of depth rather than by low-level processes related to their spatial frequency compositions. If low-level processes related to processing the spatial frequency composition of visual input affected the illusion then one would expect to see illusory effects of textures in both experiments 1 and 3.

Finally, one could argue that the presence of brick patterns in the corridor background in experiment 3 might have influenced the strength of the Poggendorff illusion by decreasing the saliency of the oblique lines. Indeed, our results revealed that reaction times tended to be slower when the central rectangle was presented on the corridor versus the uniform backgrounds. Thus, there was some evidence that there was a decrease in the saliency of the oblique lines when brick patterns were added to the background. Whether or not this could explain the decrease in illusion strength in experiment 3 is difficult to ascertain. However, our previous studies demonstrating increases in Ponzo illusion strength with the addition of pictorial depth cues would suggest otherwise [17,19,20]. Presumably, the stimuli in these studies were also less salient over busier backgrounds with more pictorial depth cues.

### 4.3. Conclusions

There are two findings from this study that deserve important consideration. First, we have not replicated findings by Talasli and Inan [3] demonstrating that brick sizes in the central rectangle invoke perceptual constancy mechanisms that would create changes in the apparent alignment of the oblique lines by changing the apparent width of the central rectangle. Second, adding pictorial depth cues seemed to decrease rather than increase the strength of the illusion. If the illusion were purely explained by low-level mechanisms, then adding pictorial cues should have not changed the strength of the illusion. In contrast, if the illusion were purely explained by cognitive-based mechanisms and driven by perceptual constancy mechanisms, then the strength of the illusion should have increased. Instead, the decrease in illusion strength in experiment 3 is suggestive that the illusion depends on both low-level and cognitive mechanisms and that deleterious effects occur on the former when the latter ascribes more certainty to the oblique lines being the same line receding into the distance.

## Data Availability

The data can be accessed here: https://osf.io/5jqdy/?view_only=af231d890b7e43b4b4536891c760777d (accessed on 1 March 2022).

## Supplementary Materials

The following supporting information can be downloaded at: https://www.mdpi.com/article/10.3390/vision6030044/s1, Video S1: Front view of a yellow rectangle placed parallel to a horizontal red line. Video S2: Front view of a yellow rectangle placed perpendicularly from a horizontal red line. Video S3: Front view of an “occluder” placed in between the horizontal lines receding in depth. Video S4: Front view of a “back-wall” placed at the termination point of two lines receding in depth.
